# Lipopeptide produced from *Bacillus* sp. W112 improves the hydrolysis of lignocellulose by specifically reducing non-productive binding of cellulases with and without CBMs

**DOI:** 10.1186/s13068-017-0993-8

**Published:** 2017-12-14

**Authors:** Jiawen Liu, Ning Zhu, Jinshui Yang, Yi Yang, Ruonan Wang, Liang Liu, Hongli Yuan

**Affiliations:** State Key Laboratory of Agrobiotechnology, College of Biological Sciences, China Agricultural University, Beijing, China

**Keywords:** Biosurfactant, *Bacillus*, Lipopeptide, Enzymatic hydrolysis, Thermostability, Lignin, Non-productive binding, Cellobiohydrolase, Carbohydrate-binding module

## Abstract

**Background:**

Surfactants have attracted increasing interest for their capability to improve the enzymatic hydrolysis of lignocellulosic biomass. Compared to chemical surfactants, biosurfactants have a broader prospect for industrial applications because they are more environmentally friendly and more effective in some researches. Commercial cellulase preparations are mainly composed of endoglucanases (EGs) and cellobiohydrolases (CBHs) that possess carbohydrate-binding modules (CBMs). However, the effects of lipopeptide-type biosurfactants on enzymatic saccharification of lignocellulose and adsorption behaviors of cellulases with CBMs remain unclear.

**Results:**

In this study, we found that *Bacillus* sp. W112 could produce a lipopeptide-type biosurfactant from untreated biomass, such as wheat bran and Jerusalem artichoke tuber. The lipopeptide could enhance the enzymatic hydrolysis of dilute acid pretreated Giant Juncao grass (DA-GJG) by fungal and bacterial enzymes. The enhancement increased over a range of temperatures from 30 to 50 °C. Lipopeptide was shown to be more effective in promoting DA-GJG saccharification than chemical surfactants at low dosages, with a best stimulatory degree of 20.8% at 2% loading of the substrates (w/w). Lipopeptide increased the thermostability of EG and CBH in commercial cellulase cocktails. Moreover, the dual effects of lipopeptide on the adsorption behaviors of cellulases were found. It specifically lowered the non-productive binding of cellulases to lignin and increased the binding of cellulases to cellulose. In addition, we investigated the influence of lipopeptide on the adsorption behaviors of CBHs with CBMs for the first time. Our results showed that lipopeptide reduced the adsorption of CBM-deleted CBH to DA-GJG to a greater extent than that of intact CBH while the non-productive binding of intact CBH to lignin was reduced more, indicating that lipopeptide decreased the binding of CBMs onto lignin but not their combination with cellulose.

**Conclusions:**

In this study, we found that lipopeptide from *Bacillus* sp. W112 promoted the enzymatic hydrolysis of DA-GJG at relative low loadings. The stimulatory effect could be attributed to increasing the cellulase thermostability, reducing non-productive adsorption of cellulases with CBMs caused by lignin and enhancing the binding of cellulases to cellulose.

**Electronic supplementary material:**

The online version of this article (10.1186/s13068-017-0993-8) contains supplementary material, which is available to authorized users.

## Background

Lignocellulose is the most abundant renewable resource on earth [1]. The hydrolysis of lignocellulosic biomass into simple sugars and subsequent fermentation to biofuels has a great meaning to energy and environmental benefits, thus attracting extensive attention of researchers [2–4]. The resistance of plant cell walls to enzymatic deconstruction largely results from their complex structure in which polysaccharides are cross-linked with the hydrophobic network of lignin [5]. Lignin removal or delocalization through pretreatment is an important and necessary step in converting lignocellulose to biofuels [6]. However, the residual lignin after pretreatment impedes enzymatic hydrolysis through obstructing enzyme–substrate proximity and causing non-productive binding of cellulases due to hydrophobic and electrostatic interaction [7–9]. Moreover,the irreversible binding to lignin hampers the recovery of enzymes and causes enzyme inactivation, thus increasing the overall cost of enzymatic hydrolysis process [10–14].

Surfactant has been one of the most common additives in the bioconversion of lignocellulose to enhance the hydrolytic performance of cellulase enzymes [15]. Chemical surfactants like PEG 6000, Tween 80 and glyceryl alcohol have been demonstrated to increase lignocellulose hydrolysis in many cases [16–19]. However, the utilization of chemical surfactants may cause pollution and reduce the ethanol productivity [20]. Secondary metabolite produced by some microorganism is a potential source of nontoxic biosurfactants that are more effective than chemical surfactants. Rhamnolipid from *Pseudomonas aeruginosa* demonstrated superior performance over Triton X-100, Tween 20 and Tween 80 in improving the glucose yield under the same condition [21, 22]. Sophorolipid from saccharomycetes increased the saccharification of oat spelt xylan and wheat bran by 20% [23]. However, these researches are limited to a few kinds of glycolipids like rhamnolipid and sophorolipid. Lipopeptide that consists of fatty acid and polypeptide has been applied in biomedical and agricultural fields due to their advantages of low toxicity and higher biodegradability and efficiency [24]. As an important category of biosurfactants, lipopeptide may also have beneficial effect on lignocellulose hydrolysis. Besides, the production of biosurfactants using glucose in most reports is cost-ineffective. Therefore, lipopeptide-producing strains that can utilize easily available biomass could advance the applications of biosurfactants in biofuel industry.

The mechanisms of enhancing the enzymatic hydrolysis of biomass by surfactants have been interpreted as increasing the stability of enzyme and reducing the non-productive adsorption caused by lignin [25–27]. Enzymes in the hydrolysis reactions are suggested to form micelles with surfactants, which relieves the denaturation caused by shearing force and heat [28]. However, the effects on enzyme stability vary with both enzyme and surfactant types [29, 30]. It has been reported that surfactants reduce the non-productive adsorption of enzymes to lignin by competing for the binding sites and the desorbed enzymes maintain active [27, 31, 32]. However, most of the researches are conducted with commercial enzyme preparations or monocomponent enzyme without carbohydrate-binding modules (CBMs) [33, 34]. CBMs are widely distributed in hydrolytic enzymes of commercial enzyme preparations derived from some fungi [35, 36]. Although CBMs can improve the hydrolysis by bringing the enzymes close to their substrates, they may also result in non-productive binding to lignin in some cases due to containing hydrophobic amino acid residues [37–39]. Rahikainen et al. [40] found that stronger lignin-binding of enzymes was detected when using *Tr*Cel7A containing CBM than Cel7A-core without CBM. Li et al. [31] have reported that the higher adsorption onto lignin of Celluclast 1.5L than that of Novozyme 188 could result from the absence of CBMs in β-glucosidase (BG) of Novozyme. As CBMs and surfactants both affect the adsorption behaviors of enzymes on lignin, it can be inferred that the cellulases with and without CBMs respond differently to surfactants. Studies on this topic will lead to a deeper understanding of the mechanisms underlying the alleviation of non-productive enzyme adsorption mediated by biosurfactants.

In our previous study, a biosurfactant-producing strain, *Bacillus* sp. W112, has been isolated from the water samples of Daqing oil field. Fourier transform infrared spectroscopy (FTIR) and thin-layer chromatography analysis showed that the biosurfactant was cyclic lipopeptide [41]. The cultivation and use of energy crop Giant Juncao grass (*Pennisetum sinese* Roxb) for cellulosic ethanol production has been studied in China for more than 20 years [42]. Here, we investigated the production of lipopeptide by *Bacillus* sp. W112 using cheap biomass and compared the effects of lipopeptide with chemical surfactants on the enzymatic hydrolysis of dilute acid pretreated Giant Juncao grass (DA-GJG). The mechanism of improving biomass hydrolysis by lipopeptide was also studied. The effects of lipopeptide on the adsorption behaviors of CBHs with and without CBMs were compared. This work contributes to developing cheap and efficient biosurfactant resources and understanding the stimulatory mechanisms of biomass saccharification by lipopeptide.

## Results

### Effects of carbon sources on lipopeptide production

**Fig. 1:**
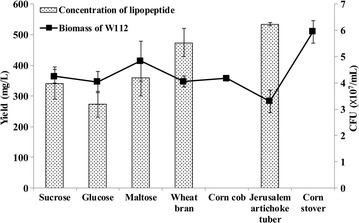
Production of lipopeptide by *Bacillus* sp. W112 using different carbon sources. The concentration of lipopeptide after fermentation was calculated according to its dry weight and the biomass of W112 was expressed by the colony forming units (CFU)

The effects of three simple sugars, sucrose, glucose and maltose and four cheap biomasses, including wheat bran, corn stover, corn cob and Jerusalem artichoke tuber, as carbon sources on lipopeptide production by *Bacillus* sp. W112 were investigated. As showed in Fig. 1, although *Bacillus* sp. W112 could utilize all seven carbon sources, it produced biosurfactant only when sucrose, glucose, maltose, wheat bran and Jerusalem artichoke tuber were used. The highest concentration of lipopeptide (535 mg/L) after fermentation was achieved with Jerusalem artichoke tuber despite that the bacterial biomass was the lowest, indicating that this cheap carbon source was suitable for lipopeptide production by *Bacillus* sp. W112. The molecular weight of lipopeptide distributed from 1046.6 Da to 1074.6 Da (see Additional file 1: Figure S1) and the critical micelle concentration was 2.19 g/L (see Additional file 2: Figure S2).

### Effect of lipopeptide addition on the enzymatic hydrolysis of DA-GJG

**Fig. 2:**
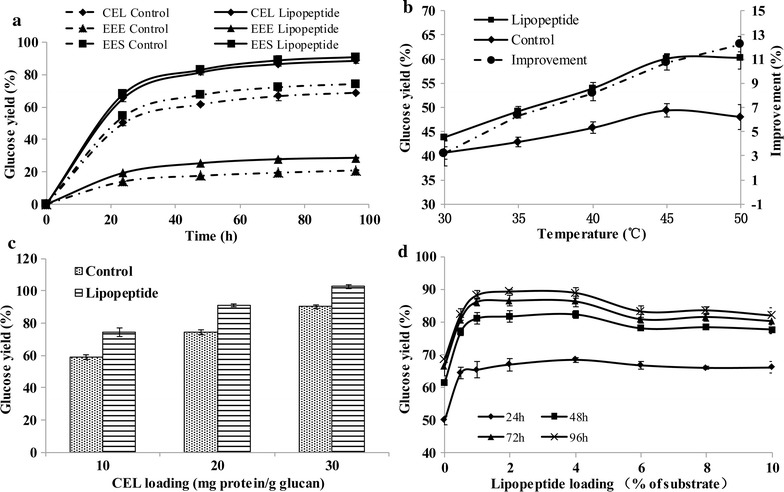
Improvement of glucose yield by lipopeptide in the enzymatic hydrolysis of DA-GJG. The improvements by lipopeptide when utilizing CEL, EES and EEE are shown in **a**. The influences of temperature, CEL loading and lipopeptide loading on the enhancement are shown in **b**–**d,** respectively. “Control” represented the system containing only substrate and enzyme. “Lipopeptide” represented the system containing lipopeptide, substrate and enzyme. Excepting indicated in the figures, the loadings of enzyme and lipopeptide were 10 mg/g glucan and 10 mg/g substrate, respectively. The temperature was 50 °C and the reducing sugars were measured after 24 h. The reducing sugar releasing was given as the glucose yield according to the cellulose content because cellulose was the main polysaccharide of DA-GJG and only cellulase was used here

The effects of enzyme sources on the stimulation of hydrolysis of DA-GJG by lipopeptide were investigated. As showed in Fig. 2a, a reducing sugar yield of 88.6% was obtained with the enzyme system of *Trichoderma longibrachiatum* commercial cellulase and beta-glucosidase (CEL) in the presence of 1% lipopeptide compared to 68.8% in the control group after 96 h. The reducing sugar yield of extracellular enzymes of wood-degrading fungus *Schizophyllum commune* (EES) [43] and extracellular enzymes of bacterial consortium EMSD5 (EEE) [44] system were 16.5 and 7.8% higher than the control group, respectively. These results indicated that lipopeptide could stimulate DA-GJG hydrolysis by enzymes of both fungal and bacterial origins.

The influence of temperature, enzyme loading and biosurfactant dosage on the stimulation effect of lipopeptide was studied with CEL. As showed in Fig. 2b, the enhancement of lipopeptide increased with temperature in the range between 30 and 50 °C from 6.3 to 12.2%. When the enzyme loadings were 10, 20 and 30 mg/g glucan, the hydrolysis improvement by lipopeptide was 15.5, 16.6 and 12.3%, respectively (Fig. 2c). It was clear that the stimulating effect of lipopeptide was prominent at various enzyme loadings. Lipopeptide promoted enzymatic hydrolysis over a wide dosage range from 0.5 to 10%, especially between 1 and 4% (Fig. 2d). The best effect was achieved with the loading of 2% and the improvement decreased when the dosage was higher.

**Fig. 3:**
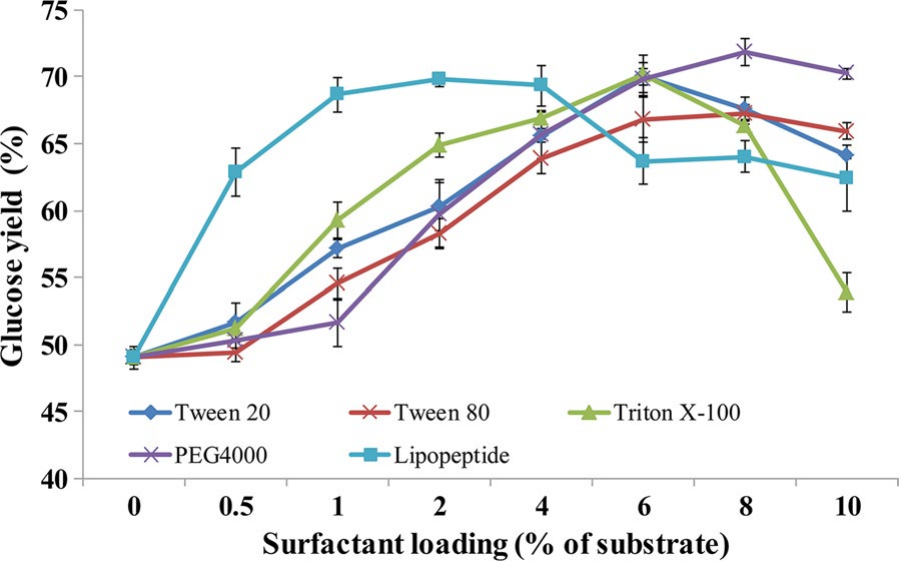
Comparing the effects of lipopeptide and four chemical surfactants with various loadings. The loadings of DA-GJG and CEL were 20 mg/mL and 10 mg/g cellulose, respectively. The temperature was 50 °C and the reducing sugars were measured after 24 h. The loadings of surfactants were indicated in the figure (w/w). The maximum hydrolysis improvement by lipopeptide was obtained with the loading of 2% but the increase showed no significant difference when comparing with that of 1% of lipopeptide

The effect of lipopeptide addition on enzymatic hydrolysis was then compared with those of commonly used chemical surfactants. All surfactants used in this study promoted DA-GJG hydrolysis at surfactant dosage between 0.5 and 10% but the dosage for optimal boosting effect was different (Fig. 3). 0.5% of lipopeptide increased the reducing sugar yield dramatically and the best stimulation of 20.8% was obtained when it was conducted at 2%. In contrast, the effects of chemical surfactants were much weaker at low concentrations and their dosage for optimal effect was above 6%. The maximum hydrolysis improvement by lipopeptide was higher than Tween 80 (*p* < 0.05) while no significant difference was observed compared with Tween 20, Triton X-100 and PEG 4000.

### Lipopeptide increases enzymatic thermostability

**Fig. 4:**
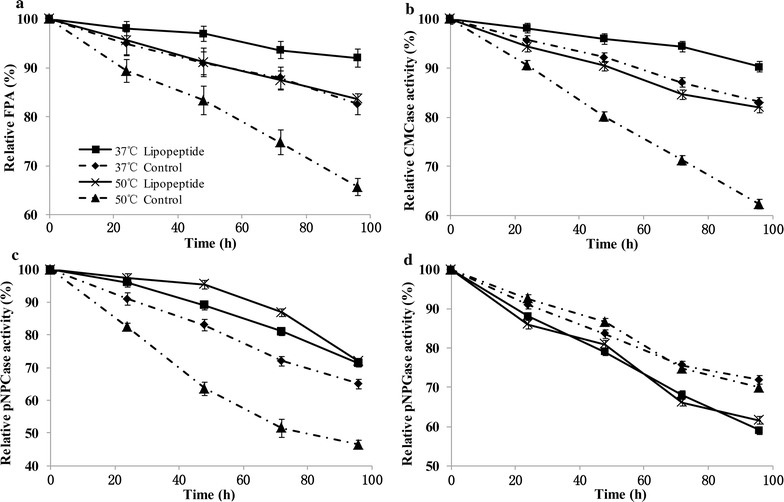
Effects of lipopeptide on the thermostability of cellulases in CEL. The overall thermostability of cellulases was expressed by FPA in **a**. The thermostability of EG (**b**), CBH (**c**) and BG (**d**) was expressed by the activities of CMCase, pNPCase and pNPGase, respectively

The effects of lipopeptide on the enzyme stability of CMCase, pNPCase, pNPGase and filter paper activity (FPA) in CEL were studied. As showed in Fig. 4a, the relative FPA decreased over time when incubated at 37 and 50 °C. After 96 h of incubation without lipopeptide, the relative FPA dropped to 83 and 66% at 37 and 50 °C, respectively. In the meanwhile, the FPA preserved 92 and 84% of its original activity in the presence of lipopeptide, indicating that lipopeptide increased the thermostability of cellulases and the effect is more remarkable at higher temperature. The relative CMCase and pNPCase activity in the presence of lipopeptide was 19.7 and 25.5% higher than that of control at 50 °C after 96 h, respectively (Fig. 4b, c). However, the relative pNPGase activity decreased slightly with the addition of lipopeptide (Fig. 4d). These results showed that lipopeptide enhanced the thermostability of EG and CBH but showed no benefits to that of BG.

The interaction between lipopeptide and enzyme was further studied by fluorescence with pyrene probe, where enzyme molecules would lower the ratio of the first and third peaks (*I*_1_/*I*_3_) of pyrene if they bind with surfactant [28]. It could be inferred that lipopeptide combined with enzyme directly and formed micelles together, which contributed to improving the thermostability of CEL (see Additional file 3: Figure S3).

### Lipopeptide reduces non-productive binding of enzyme to lignin

**Fig. 5:**
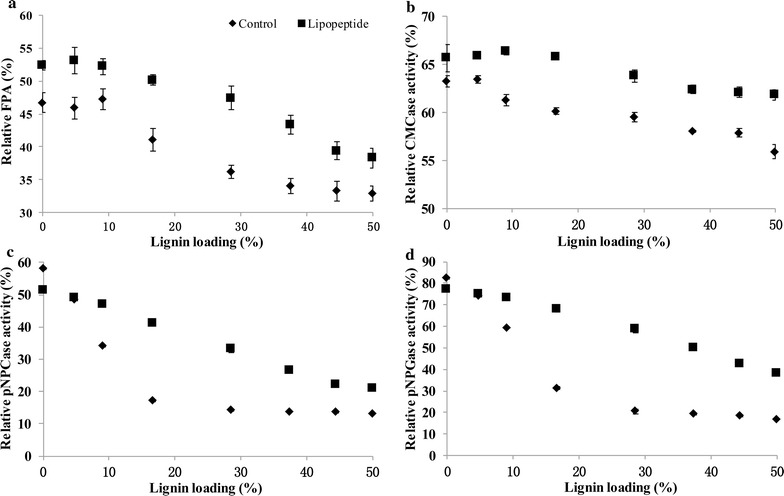
Effects of lipopeptide on the adsorption behaviors of CEL. The substrates contained 20 mg of Avicel and various proportions (w/w) of lignin. The proportions of free cellulases (**a**), EG (**b**), CBH (**c**) and BG (**d**) were expressed by their respective activities

**Fig. 6:**
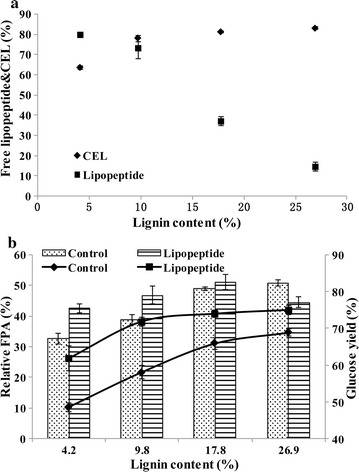
Adsorption of lipopeptide and CEL on DA-GJGs with different lignin contents. **a** The adsorption of lipopeptide and CEL on DA-GJG and DA-GJG-DLs was measured by Bradford protein assay kit [45]. **b** Displayed the effect of lipopeptide on the hydrolysis and CEL adsorption onto DA-GJG and DA-GJG-DLs. Columns and lines indicated FPA in the supernatant and glucose yield, respectively. “Control” represented the system containing only substrate and enzyme. “Lipopeptide” represented the system containing lipopeptide, substrate and enzyme

To investigate the effect of lipopeptide on the adsorption of cellulases to cellulose and lignin, the cellulase activities of commercial preparation CEL in the presence of microcrystalline cellulose (Avicel PH-101) with increasing lignin addition were determined. As showed in Fig. 5, the relative activities of CMCase, pNPCase, pNPGase and FPA in the supernatant with lipopeptide were all higher than that of control, which demonstrated that lipopeptide increased the proportion of free cellulases. The enhancement was more prominent when the lignin content exceeded 5% (w/w) of the substrate. For example, no significant influence of lipopeptide on pNPCase activity was observed with 4.8% of lignin. However, the relative activity of pNPCase increased by 19.0% in the presence of lipopeptide when the substrate contained 28.6% lignin (w/w) (Fig. 5c). Also, more lipopeptide adsorption to substrate was found when the lignin content was higher, suggesting a stronger affinity of lipopeptide towards lignin than cellulose (Fig. 6a). These results suggested that lipopeptide increased the concentration of free enzymes mainly through reducing non-productive binding caused by lignin via competing for the binding sites. Interestingly, lipopeptide decreased the activities of pNPCase and pNPGase in the supernatant when no lignin was added to Avicel (Fig. 5c, d). We also conducted the adsorption experiment using DA-GJG with different lignin contents and found that lipopeptide reduced the FPA in the supernatant when the lignin content was low (Fig. 6b). These results indicated that lipopeptide improved the adsorption of cellulases to cellulose.

### Effect of lipopeptide on CBM-containing CBH adsorption

CBM plays an important role in the binding of enzyme to polysaccharides [46]. Since lipopeptide and CBMs both affect the adsorption behaviors of enzymes significantly, the effect of lipopeptide on cellulases with CBMs was investigated. The genes of 45818-WT (GenBank accession number: MF802278), a CBH containing three CBMs, and 45818-Core, the catalytic domain of 45818-WT without CBMs, were cloned from the metagenome of microbial consortium EMSD5 [44] and expressed in *E. coli* BL21 (see Additional file 4: Figure S4). The response of CBMs to the effect of lipopeptide was studied using 45818-WT and 45818-Core.

**Fig. 7:**
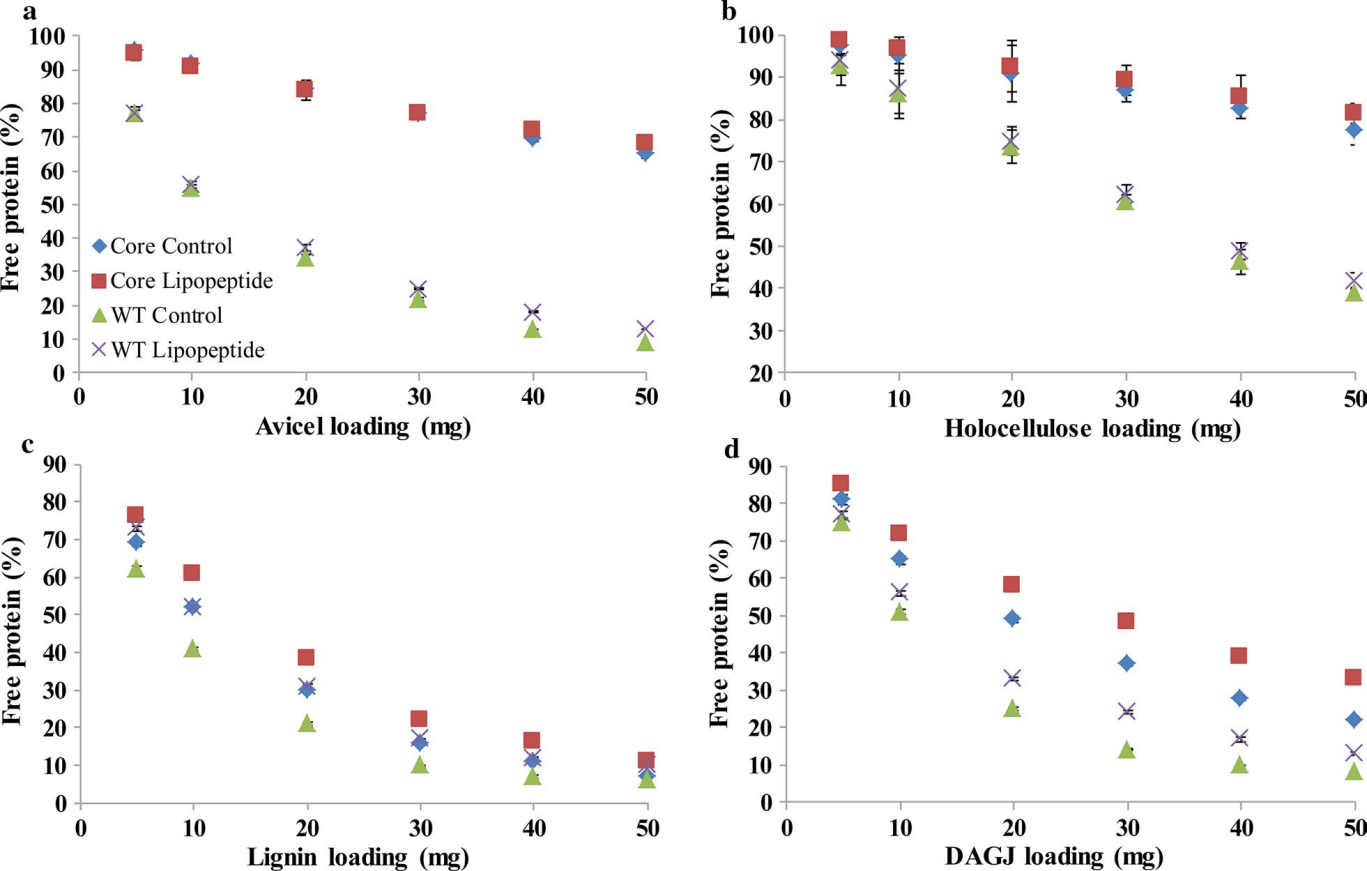
Effects of lipopeptide on the fractions of free 45818-WT and 45818-Core. Avicel (**a**), holocellulose (**b**), lignin (**c**) and DA-GJG (**d**) were used as substrate, respectively. The pNPCase activities in the supernatant were measured and the concentration could be figured out according to the standard curve (see Additional file 7: Figue S7)

As showed in Fig. 7a, 45818-WT revealed stronger affinity to Avicel than 45818-Core which indicated that CBMs of 45818-WT were beneficial to CBH binding to microcrystalline cellulose. Lipopeptide showed no significant effect on the affinity of the two CBHs to cellulose. 45818-WT and 45818-Core bound to holocellulose even less than to Avicel (Fig. 7b) and lipopeptide did not release CBHs from holocellulose as well. These results suggested that lipopeptide hardly influenced the binding of CBHs to cellulose and hemicellulose. When lignin was used as the substrate, the affinity of 45818-WT to lignin was stronger than that of 45818-Core. For example, 48% of 45818-Core but 59% of 45818-WT were adsorbed by 10 mg of lignin, respectively. Moreover, the fractions of free 45818-WT and 45818-Core were increased with the addition of lipopeptide (Fig. 7c). This indicated that CBMs indeed resulted in more enzyme adsorption to lignin and lipopeptide reduced this non-productive binding. Lipopeptide also increased the fractions of both free 45818-WT and 45818-Core with the substrate of DA-GJG (Fig. 7d). Considering previous results, we could infer that the improvement of free CBHs on DA-GJG mainly resulted from the reduction of binding to lignin rather than cellulose.

To quantify the effects of lipopeptide on the adsorption behaviors of 45818-WT and 45818-Core, the amounts of free and adsorbed enzymes were measured and fitted to the Langmuir adsorption isotherm (see Additional file 5: Figure S5). As showed in Table 1, all the data fitted to Langmuir adsorption isotherm well (*R* ^2^ > 0.95) except that of 45818-Core on Avicel. The *E*_max_ (maximum adsorbed enzymes) and *K*_p_ (equilibrium adsorption constant) of 45818-WT on lignin in control group were 141.96 nmol/g and 2.02 L/μmol, respectively. However, the *E*_max_ and *K*_p_ decreased to 102.27 nmol/g and 1.17 L/μmol when lipopeptide was used. This indicated that lipopeptide lowered the maximum adsorption and affinity of 45818-WT to lignin. As for 45818-Core, lipopeptide decreased the *E*_max_ and *K*_p_ on lignin from 101.47 nmol/g and 1.19 L/μmol to 87.32 nmol/g and 0.86 L/μmol, respectively. But lipopeptide showed no significant effects on *E*_max_ and *K*_p_ on Avicel. These results also indicated that lipopeptide specifically reduced the adsorption of enzymes onto lignin.

**Table 1:** Effects of lipopeptide on the Langmuir adsorption parameters of 45818-WT and 45818-Core on different substrates

Substrate	Enzyme	Lipopeptide	*E*_max_ (nmol/g)	*K*_p_ (L/μmol)	*R* ^2^
Avicel	WT	No	98.82 ± 5.71	1.13 ± 0.16	0.985
	WT	Yes	95.68 ± 2.55	1.12 ± 0.07	0.997
Lignin	WT	No	141.96 ± 5.44	2.02 ± 0.22	0.990
	WT	Yes	102.27 ± 3.89	1.17 ± 0.11	0.993
DA-GJG	WT	No	128.39 ± 8.20	1.74 ± 0.31	0.972
	WT	Yes	99.25 ± 2.08	1.07 ± 0.05	0.998
Lignin	Core	No	101.47 ± 4.33	1.19 ± 0.13	0.991
	Core	Yes	87.32 ± 10.09	0.86 ± 0.23	0.956
DA-GJG	Core	No	86.44 ± 12.13	0.65 ± 0.19	0.957
	Core	Yes	65.40 ± 5.84	0.51 ± 0.09	0.987

Lipopeptide decreased the *E*_max_ of 45818-WT and 45818-Core by 28.0 and 14.1% on lignin and by 22.7 and 24.3% on DA-GJG, respectively. Apparently, *E*_max_ of 45818-WT was decreased more on lignin while less on DA-GJG than 45818-Core. Similarly, *K*_p_ of 45818-WT was decreased more on lignin but less on DA-GJG when lipopeptide was added. This indicated that lipopeptide released more 45818-WT from lignin while more 45818-Core from DA-GJG.

## Discussion

*Bacillus* sp. W112 produced lipopeptide using cheap carbon source such as wheat bran and Jerusalem artichoke tuber, demonstrating its broader prospect for industrial applications than strains that utilized glucose. Increasing effect of lipopeptide on the enzymatic hydrolysis of DA-GJG was first found in this study. Although the stimulation of biomass hydrolysis by biosurfactants has been reported in other researches [21–23, 47, 48], these experiments were carried out only with a certain concentration of biosurfactant. We compared the effects of lipopeptide with chemical surfactants at various loadings ranging from 0.5 to 10% and found that the enhancement by the low-level (2%) lipopeptide was as strong as that of high-level (> 6%) chemical surfactants (Fig. 3), which was more environment-friendly and beneficial to the fermentation [49].

The improvement of reducing sugar yield by lipopeptide increased with temperature (Fig. 2b), which suggests that lipopeptide performs optimally under thermophilic conditions. We also found that the influence of lipopeptide on enzyme thermostability varied depending on the enzyme types. In the presence of lipopeptide, enzyme inactivation was relieved for EG and CBH but was aggravated slightly for BG (Fig. 4). The different effects on enzyme stability also have been observed when it comes to chemical surfactants [29]. It was found that trehalose lipid protected bovine serum albumin (BSA) from thermal denaturation but promoted thermal unfolding of cytochrome c [50]. Zou et al. [51] reported that the hydrophobic interaction and hydrogen bonds played an important role in surfactant binding to BSA. Therefore, the interaction between enzymes and surfactants is essential for altering the stability of proteins [28, 52], which is affected by the structure, surface residues and even concentrations of enzymes [16]. The difference between EG, CBH and BG in CEL probably leaded to the increase in thermostability of EG and CBH but the denaturation of BG. However, the enzyme stability is not necessarily the same as hydrolysis ability. For example, while its boosting effect on lignocellulose hydrolysis was specific for CBH, PEG3000 increased the thermostability of EG but not CBH, which could be interpreted as increasing in water availability [30]. The actual effects of surfactants result from comprehensive factors including enzyme stability, substrate accessibility and so on.

Lipopeptide tended to bind with lignin rather than cellulose while cellulases were opposite (Fig. 6a). It could explain why lipopeptide lowered the adsorption of cellulases to lignin but not cellulose. The lower optimal concentration of lipopeptide than chemical surfactants possibly ascribes to the high specificity of reducing lignin-binding (Fig. 3). Although most researches focused on the ability of surfactants to reduce the non-productive adsorption, some reports showed that surfactants also enhanced the combination of enzymes with substrates [53, 54]. However, they did not explain why surfactants increased the binding to substrate under certain conditions but reduced the adsorption under others. The hydrogen bonding between cellulose chains can be replaced by water molecules, which possibly promotes the swelling of cellulose [55]. Surfactants may contribute to disturbing hydrogen bonding and thus make cellulose more accessible to cellulases [56]. Lipopeptide increased the proportions of free cellulases only when the substrate contained lignin but decreased the free cellulases with the substrate of cellulose (Figs. 5c, d, 6b). Considering previous reports and the results in this work, it can be concluded that lipopeptide has a dual effect on the adsorption behaviors of cellulases. On the one hand, it reduces the lignin-binding of enzymes; on the other hand, it could be speculated that lipopeptide promotes the expansion of cellulose and more binding sites are exposed which results in more adsorption of cellulases to cellulose. The two effects exist at the same time but the previous one is dominant and the influence of lipopeptide observed depends on the lignin content in the substrate. When the substrate contains a high amount of lignin, the addition of lipopeptide will result in reduction of non-productive adsorption and increase of free enzymes. In contrast, when it contains no or a low amount of lignin, the adsorption caused by lignin is insignificant and lipopeptide enhanced the binding to cellulose thus reducing the free enzymes. This may be one of the reasons that surfactant significantly enhanced the conversion of substrate with little lignin-binding in this study (Fig. 6b) and other report [57]. Lipopeptide did not increase the adsorbed CBHs with the substrate of Avicel might result from the concentrations and structures of 45818-WT and 45818-Core were different from CEL in those experiments (Fig. 7a, b and Table 1).

CBMs are common in cellulases and improve the hydrolytic efficiency via targeted binding to cellulose [37]. The role of CBMs at the existence of surfactants was investigated for the first time. Irrespective of the presence of CBMs, lipopeptide specifically reduced the lignin-binding. However, lipopeptide increased more dissociative 45818-WT with lignin but less with DA-GJG than 45818-Core. The different effects of lipopeptide on adsorption of the two CBHs can be interpreted as the varying role of the CBMs. When lignin was used as the substrate, CBMs caused more lignin-binding because of hydrophobic interaction [38, 39] so lipopeptide reduced more non-specific binding of 45818-WT than 45818-Core. When the DA-GJG was used as substrate, CBMs promoted the combination of cellulases with cellulose more than lignin. Therefore, more 45818-WT was binding to cellulose than 45818-Core. As lipopeptide only reduces the adsorption caused by lignin, the increase of free 45818-WT was weaker than that of 45818-Core. The effect of lipopeptide on CBMs is highly specific for reducing non-productive adsorption to lignin while not decreasing the combination of CBMs with cellulose, indicating that lipopeptide is suitable for cellulases with CBMs. These results also suggest that the practical effects of surfactants depend on many factors like types of substrates and enzymes.

In summary, we studied the effects of lipopeptide on the enzymatic hydrolysis of lignocellulose for the first time. It was found that lipopeptide could improve the conversion of DA-GJG and the stimulation was more prominent than that of chemical surfactants at low dosages. The mechanisms of lipopeptide for improving enzymatic conversion could be interpreted as follows: (1) lipopeptide improves the stability of EG and CBH; (2) lipopeptide reduces non-productive adsorption of both catalytic domain and CBM to lignin specifically; (3) lipopeptide promotes the combination of cellulases with cellulose. The ratio of free/adsorbed cellulases is influenced by the dual effects of lipopeptide.

## Conclusions

Lipopeptide produced by *Bacillus* sp. W112 with cheap carbon sources enhanced the enzymatic conversion of DA-GJG by various cellulases and showed stronger stimulation at low concentrations. Lipopeptide relieved the denaturation of cellulases by improving the thermostability of EG and CBH. The dual effects of reducing non-productive binding to lignin and enhancing the combination of cellulases with cellulose were observed. Lipopeptide affected differently the adsorption behaviors of cellulases with CBMs depending on the role of CBMs. When the CBMs caused non-productive binding, the desorption of enzymes containing CBMs was stronger than those without CBMs. If CBMs promoted the binding to cellulose, the desorption of enzymes with CBMs was less prominent.

## Methods

### Lipopeptide preparation

*Bacillus* sp. W112 was cultivated at 37 °C for 12 h in LB medium. 7.5 mL (5%, v/v) of inoculum was inoculated into a 500 mL erlenmeyer flask containing 150 mL of fermentation medium. The composition of the fermentation medium was listed as follows: carbon source 30 g/L, K_2_HPO_4_ 1 g/L, KH_2_PO_4_ 1 g/L, NaNO_3_ 1 g/L, (NH_4_)_2_SO_4_ 0.5 g/L, yeast extract powders 0.2 g/L, MgSO_4_·7H_2_O 0.2 g/L, CaCl_2_ 0.01 g/L, MnSO_4_ 0.01 g/L, FeSO_4_ trace. Wheat bran, corn stover, corn cob and Jerusalem artichoke tuber were ground to 70 meshes. After cultivating at 37 °C for 24 h, supernatant was collected by centrifugation at 4 °C, 10,000 rpm for 10 min. Lipopeptide was collected by acid precipitation method [58]. The biomass of *Bacillus* sp. W112 was measured using dilution-plate method [59].

### Molecular weight and critical micelle concentration of lipopeptide

The lipopeptide was dissolved in methanol to measure the molecular weight by liquid chromatography combined with mass spectrum (LC–MS) with a Thermo Q-exactive high-resolution mass spectrometer (Thermo Scientific, Waltham, MA, USA).

The lipopeptide solutions with different concentrations (0.2–20 mg/mL) were prepared, whose surface tension was measured by drop weight method [60]. The critical micelle concentration of lipopeptide was calculated according to the surface tension.

### Substrates and enzymes preparation

Giant Juncao grass was kindly provided by Chongqing City Construction Investment Co. Ltd. The grass was ground to 70 meshes and then treated with dilute acid as described elsewhere [43]. The treated solid residue was collected using filter papers and washed thoroughly with deionized water. Finally, the DA-GJG powders were dried at 50 °C. For the experiments showed in Fig. 6, the DA-GJG was also delignified to different extent using sodium chlorite [61]. The main components of Giant Juncao grass before and after pretreatment are showed in Table 2.

**Table 2:** The main components of Giant Juncao grass before and after pretreatment

	Cellulose (%)	Hemicellulose (%)	Klason lignin (%)
Before pretreatment	39.9 ± 0.6	20.8 ± 0.3	20.7 ± 0.2
DA-GJG	46.1 ± 0.6	15.0 ± 0.7	26.9 ± 2.4
DA-GJG-DL1	58.2 ± 2.5	13.4 ± 0.3	17.8 ± 0.3
DA-GJG-DL2	67.7 ± 2.0	12.6 ± 0.2	9.8 ± 0.7
DA-GJG-DL3	76.6 ± 0.3	11.3 ± 0.2	4.1 ± 0.1

DA-GJG-DL represents the DA-GJG treating with sodium chlorite to delignify. The numbers at the end indicate the times of delignification. DA-GJG-DL was only utilized in the experiments as shown in Fig. 6. The compositional analysis was conducted as described elsewhere [43]

Lignin was kindly provided by Shandong Longlive Biotechnology Co, Ltd. This commercial lignin powder was isolated from corn cob residues by alkaline extraction and then purified by acid precipitation, which indicated that it was Kraft lignin. FTIR analysis also showed the characters of HGS lignin (see Additional file 6: Figure S6) [62]. Avicel PH101 (11365) and cellulase (C9748) were purchased from Sigma. Commercial BG (TE561) was purchased from Beijing Huajing Technology Co, Ltd. The methods of extracting EES and EEE were described elsewhere [43, 44].

The gene sequence of CBH (ID: 45818) comes from the metagenome of EMSD5 [44]. We obtained the gene segments of 45818-WT and 45818-Core by PCR and the primers are shown in Table 3. The genes were inserted into pET30a vector and expressed in *E. coli* BL21. The recombinant proteins were purified with a nickel column.

**Table 3:** Primers used in this work

Type	Sequence (from 5′ to 3′)		Restriction enzyme
45818-WT	Forward	CGCGGATCCACACTGCAGTCTAATCTGGTTGTAA	*Bam*HӀ
	Reverse	CCGCTCGAGTTAAATTTCTGTTCCGCCGTAAGTA	*Xho*Ӏ
45818-Core	Forward	CGCGGATCCAAAGAGGAAAATGACATCGTTCC	*Bam*HӀ
	Reverse	CCGCTCGAGATGAGTTACTTTAATGCCATTTGTA	*Xho*Ӏ

### Enzymatic hydrolysis of DA-GJG

The experiment was conducted in sodium acetate buffer (50 mM, pH 5.0) with a total volume of 1 mL. Substrate (2%, w/v) was incubated with commercial cellulase (10 mg protein/g glucan) and BG (2 mg protein/g glucan) at 50 °C, 200 rpm. The dosage of surfactant was 1% (w/w) of the substrate. After 24 h, the reducing sugars were quantified by the dinitrosalicylic acid (DNS) assay [63].

### Thermostability of CEL

CEL and lipopeptide (no addition in control group) were dissolved in 1 mL sodium acetate buffer (50 mM, pH 5.0) and incubated at 37 or 50 °C, 200 rpm. The FPA, CMCase (on behalf of EG), pNPCase (on behalf of CBH) and pNPGase (on behalf of BG) were measured every 24 h. The relative activities were calculated according to the initial activities which were designated as 100%. No significant influence of lipopeptide on the determination of the four activities was observed.

### Correlation between cellulase activities and enzyme loadings

The FPA and activities of CMCase, pNPCase, pNPGase were assayed as described elsewhere [43, 64]. The protein concentration measured directly in supernatant does not represent actual enzyme concentration because of the interference of lipopeptide. A positive correlation between the activities and concentrations of enzymes was found (see Additional file 7: Figure S7), so the relative concentrations of free enzymes could be expressed by their relative activities. In terms of CBH and BG, the actual concentrations could be figured out according to the standard curve and corresponding activities. Lipopeptide showed no significant effect on the determination of these four activities under the condition here (data not showed).

### Adsorption behaviors of CEL

Substrate (2% of DA-GJG with different lignin contents or Avicel and lignin), CEL and lipopeptide (no addition in control group) were mixed in 1 mL of sodium acetate buffer (50 mM, pH 5.0) and incubated at 0 °C, 200 rpm for 1 h. The activities of CMCase, pNPCase, pNPGase and FPA in the supernatant were measured after incubation [43, 64]. The activities in the system without any substrates were designated as 100%. The proportions of free enzymes were expressed by their relative activities in this study (see Additional file 7: Figure S7).

### Adsorption of CBHs and isotherms

Lipopeptide (no addition in control group) and CBH (0.5 μmol/L of 45818-WT or 45818-Core) were mixed with four kinds of substrates (Avicel, holocellulose from Giant Juncao grass, lignin and DA-GJG), respectively, in 1 mL of sodium acetate buffer (50 mM, pH 5.0) and incubated at 0 °C, 200 rpm for 1 h. The activity of pNPCase in the supernatant was measured after incubation. The concentrations of the CBHs were calculated according to the standard curve (see Additional file 7).

For adsorption isotherms researches, the dosages of substrates (10 mg) and lipopeptide (0.1 mg) were constant and the loadings of CBHs were gradient (0.5/1.0/1.5/2.0/2.5/3.0 μM). The concentrations of free CBHs were measured after incubation as mentioned above. The amounts of free and adsorbed CBHs were fitted to the Langmuir adsorption isotherm using OriginPro 9.0.

## Additional files

**Additional file 1: Figure S1.** LC–MS analysis of lipopeptide. The peaks with m/z ratios of 523.8, 530.8 and 537.8 suggested that lipopeptide was a mixture of three homologs containing fatty acid chains with different lengths.

**Additional file 2: Figure S2.** The critical micelle concentration of lipopeptide. The surface tension of lipopeptide solution with different concentrations was showed here. The sudden change in the slope of the surface tension vs concentration curve indicated the critical micelle concentration of lipopeptide.

**Additional file 3: Figure S3.** Effects of lipopeptide and enzyme on the I_1_/I_3_ ratio of pyrene. The experimental design was described elsewhere [28]. In briefly, lipopeptide and CEL were dissolved with saturated solution of pyrene. The fluorescence intensity was detected and compared with the system containing no CEL. Evolution of I_1_/I_3_ ratio could reflect the interaction between lipopeptide and enzyme.

**Additional file 4: Figure S4.** Modular representation (a) and SDS-PAGE analysis (b) of 45818-WT and 45818-Core. Theoretical molecular weights of 45818-WT and 45818-Core are 102.9 kD and 76.5 kD, respectively.

**Additional file 5: Figure S5.** Adsorption isotherms of 45818-WT and 45818-Core on Avicel (a), DA-GJG (b) and lignin (c).

**Additional file 6: Figure S6.** FTIR spectra of commercial lignin. The lignin was pretreated by mixing with KBr and grinding. The absorption bands at 834 cm^−1^ (C-H out of plane in positions 2 and 6 of S units), 1129 cm^−1^ (typical aromatic C-H bending in-plane for S units) and 1167 cm^−1^ (C=O in ester groups (conjugated), typical for HGS lignin) indicated the features of HGS lignin [62].

**Additional file 7: Figure S7.** Correlation between FPA (a), activities of CMCase (b), pNPCase (c), pNPGase (d) and enzyme loading. One unit of enzymatic activity was defined as the amount of enzyme that produced 1 μmol of glucose (for FPA and CMCase) or pNP (for pNPCase and pNPGase) per minute under the conditions.

## Abbreviations

CBM: carbohydrate-binding module
EG: endoglucanase
CBH: cellobiohydrolase
BG: β-glucosidase
FTIR: Fourier transform infrared spectroscopy
DA-GJG: dilute acid pretreated Giant Juncao grass
CFU: colony forming units
CEL: commercial cellulase and β-glucosidase
EES: extracellular enzymes of *Schizophyllum commune*
EEE: extracellular enzymes of EMSD5
CMC: carboxymethyl cellulose
pNPC: *p*-nitrophenyl-d-cellobioside
pNPG: *p*-nitrophenyl-d-glucopyranoside
FPA: activity of filter paper
BSA: bovine serum albumin
LC–MS: liquid chromatography combined with mass spectrum
PCR: polymerase chain reaction
DNS: 3,5-dinitrosalicylic acid
