# Supplementary figures and images for: Lipopeptide produced from Bacillus sp. W112 improves the hydrolysis of lignocellulose by specifically reducing non-productive binding of cellulases with and without CBMs
Source: Biotechnol Biofuels. 2017 Dec 14;10:301. doi: 10.1186/s13068-017-0993-8 (PMC5729243; doi:10.1186/s13068-017-0993-8)

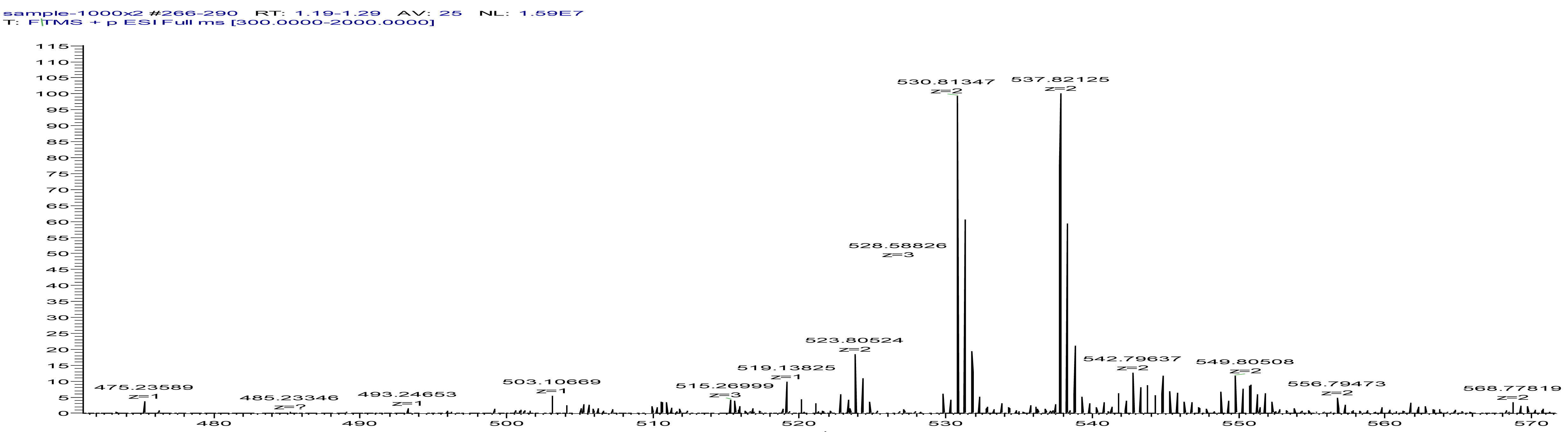

Supplement: Supplementary file 1 — Additional file 1: Figure S1. LC–MS analysis of lipopeptide. The peaks with m/z ratios of 523.8, 530.8 and 537.8 suggested that lipopeptide was a mixture of three homologs containing fatty acid chains with different lengths. [file 13068_2017_993_MOESM1_ESM.tif]

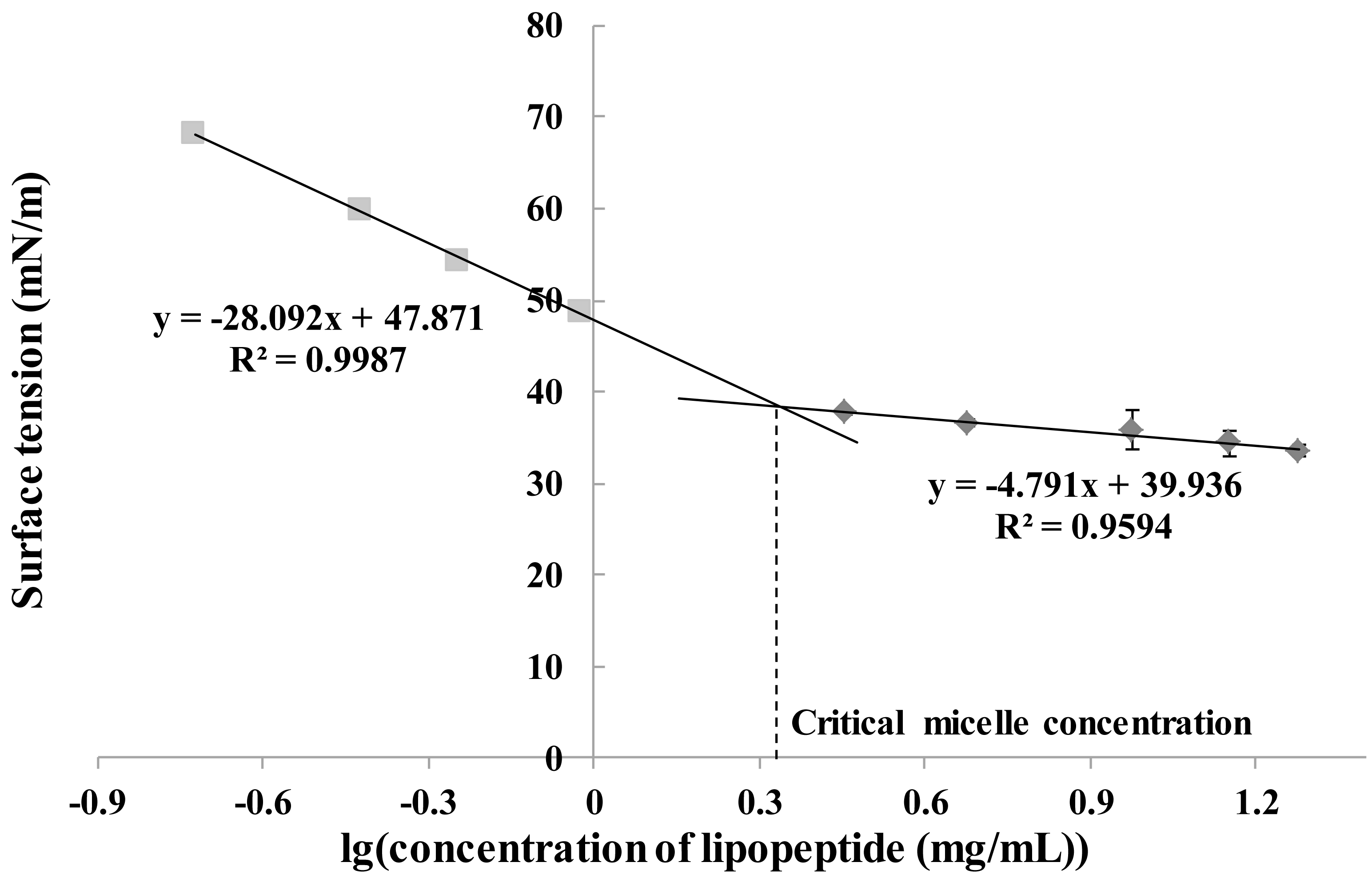

Supplement: Supplementary file 2 — Additional file 2: Figure S2. The critical micelle concentration of lipopeptide. The surface tension of lipopeptide solution with different concentrations was showed here. The sudden change in the slope of the surface tension vs concentration curve indicated the critical micelle concentration of lipopeptide. [file 13068_2017_993_MOESM2_ESM.tif]

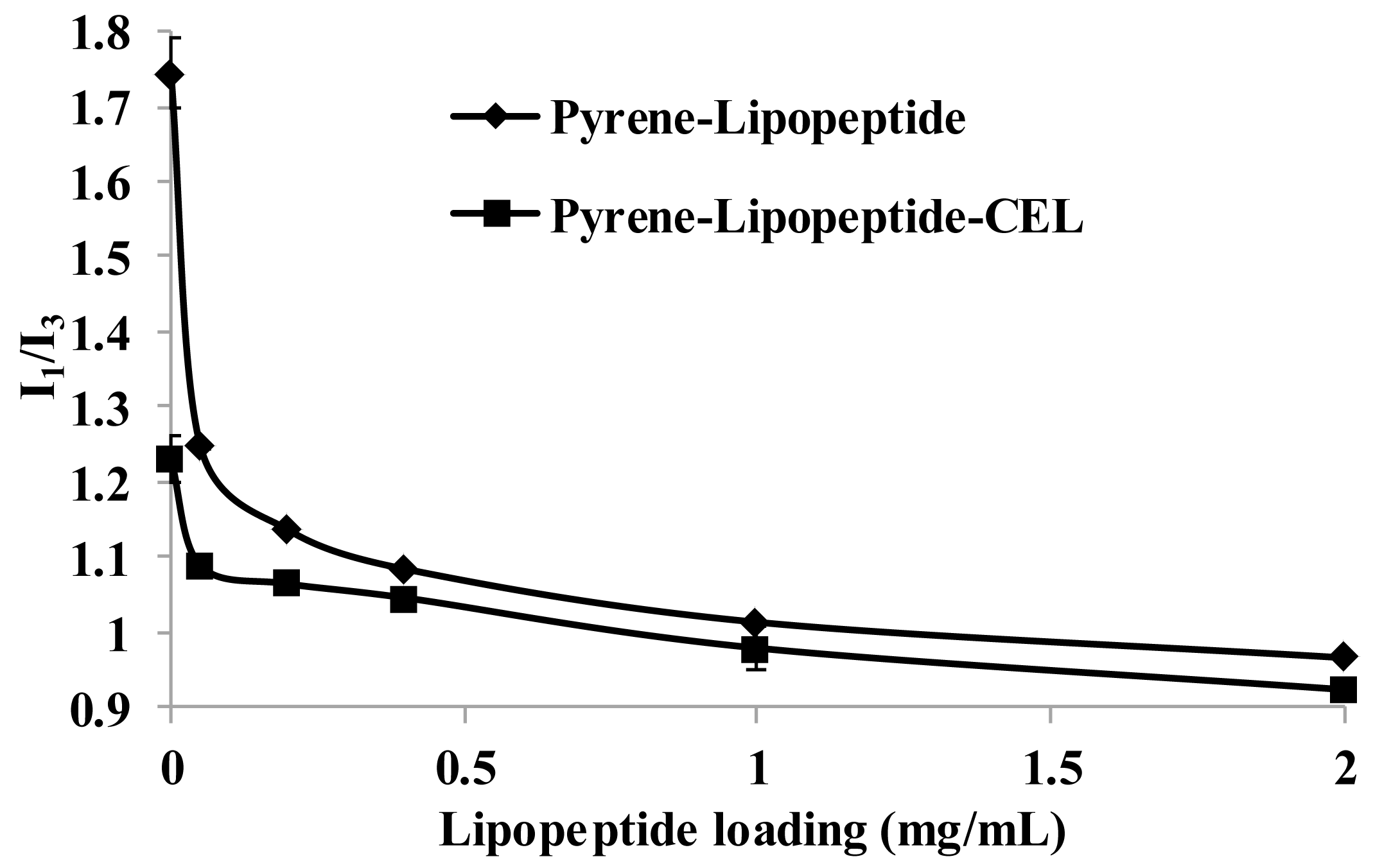

Supplement: Supplementary file 3 — Additional file 3: Figure S3. Effects of lipopeptide and enzyme on the I1/I3 ratio of pyrene. The experimental design was described elsewhere [28]. In briefly, lipopeptide and CEL were dissolved with saturated solution of pyrene. The fluorescence intensity was detected and compared with the system containing no CEL. Evolution of I1/I3 ratio could reflect the interaction between lipopeptide and enzyme. [file 13068_2017_993_MOESM3_ESM.tif]

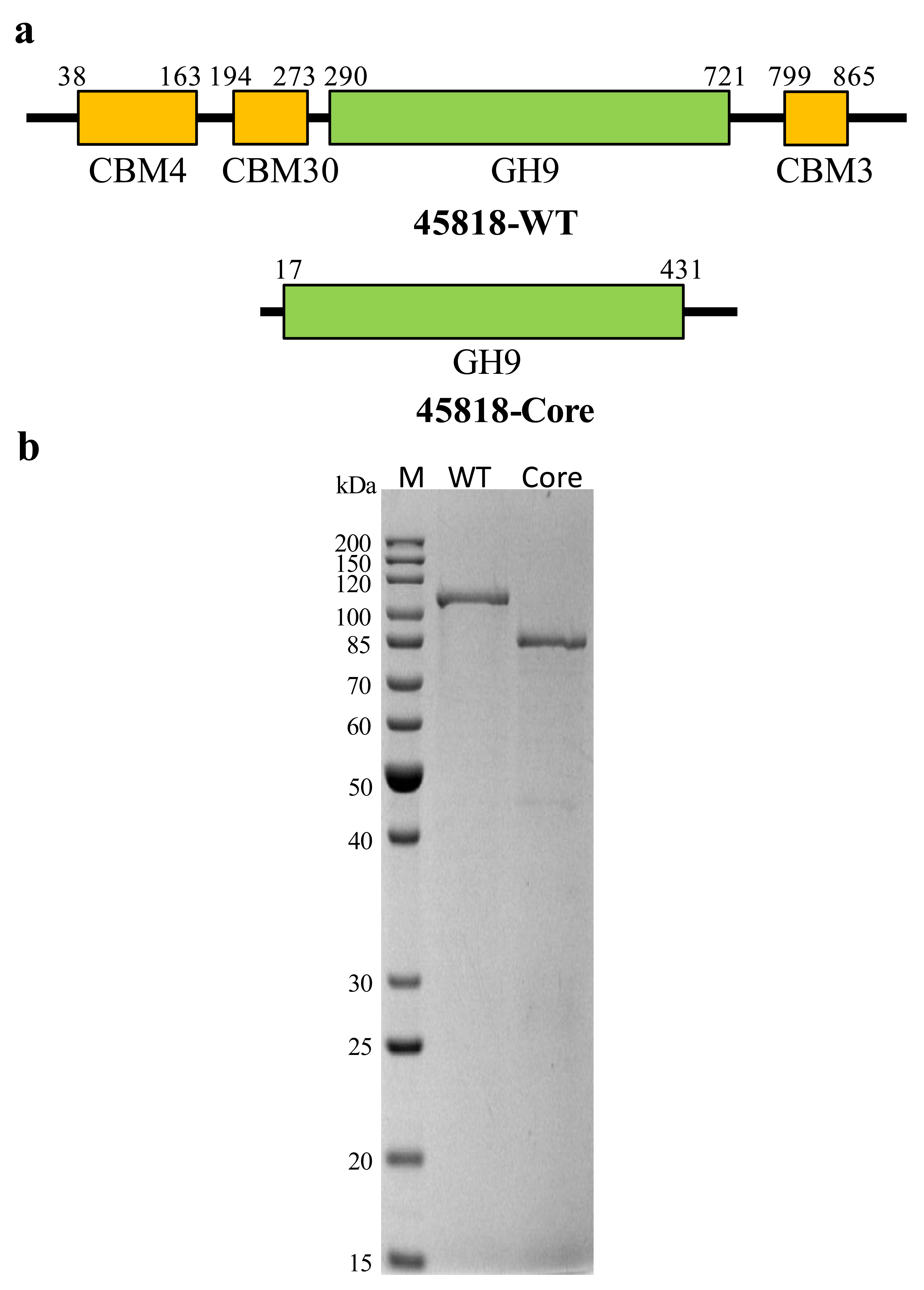

Supplement: Supplementary file 4 — Additional file 4: Figure S4. Modular representation (a) and SDS-PAGE analysis (b) of 45818-WT and 45818-Core. Theoretical molecular weights of 45818-WT and 45818-Core are 102.9 kD and 76.5 kD, respectively. [file 13068_2017_993_MOESM4_ESM.tif]

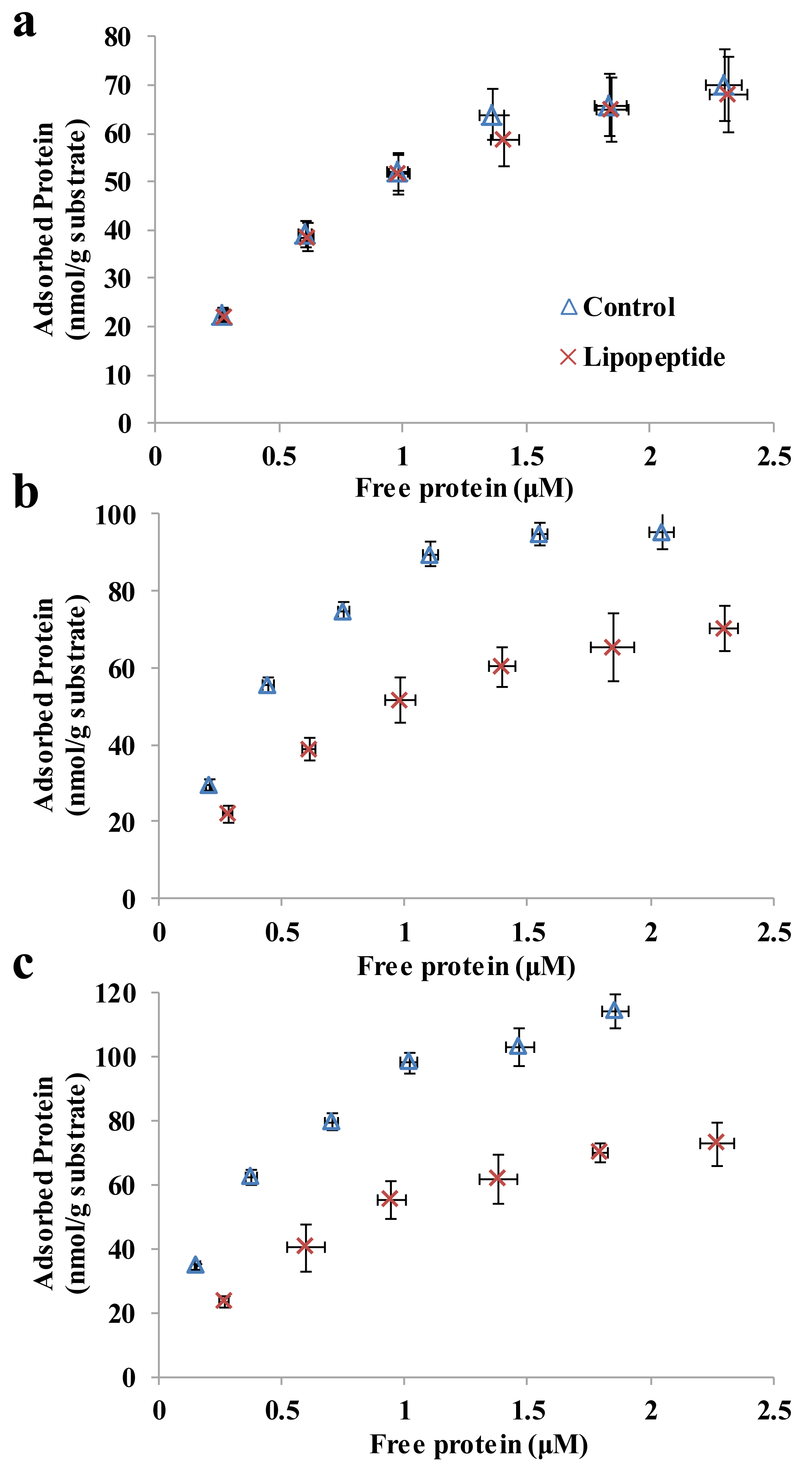

Supplement: Supplementary file 5 — Additional file 5: Figure S5. Adsorption isotherms of 45818-WT and 45818-Core on Avicel (a), DA-GJG (b) and lignin (c). [file 13068_2017_993_MOESM5_ESM.tif]

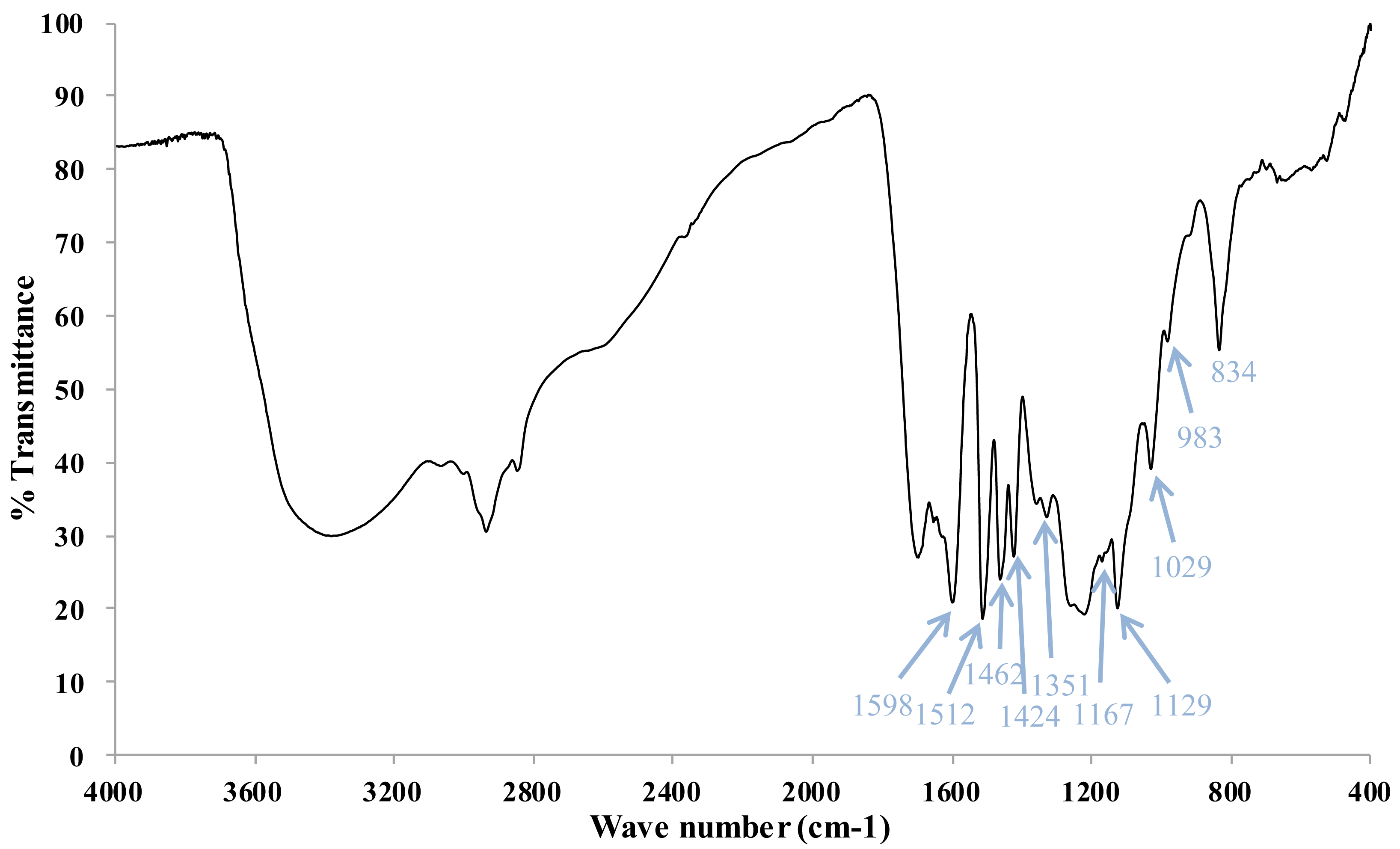

Supplement: Supplementary file 6 — Additional file 6: Figure S6. FTIR spectra of commercial lignin. The lignin was pretreated by mixing with KBr and grinding. The absorption bands at 834 cm−1 (C-H out of plane in positions 2 and 6 of S units), 1129 cm−1 (typical aromatic C-H bending in-plane for S units) and 1167 cm−1 (C=O in ester groups (conjugated), typical for HGS lignin) indicated the features of HGS lignin [62]. [file 13068_2017_993_MOESM6_ESM.tif]

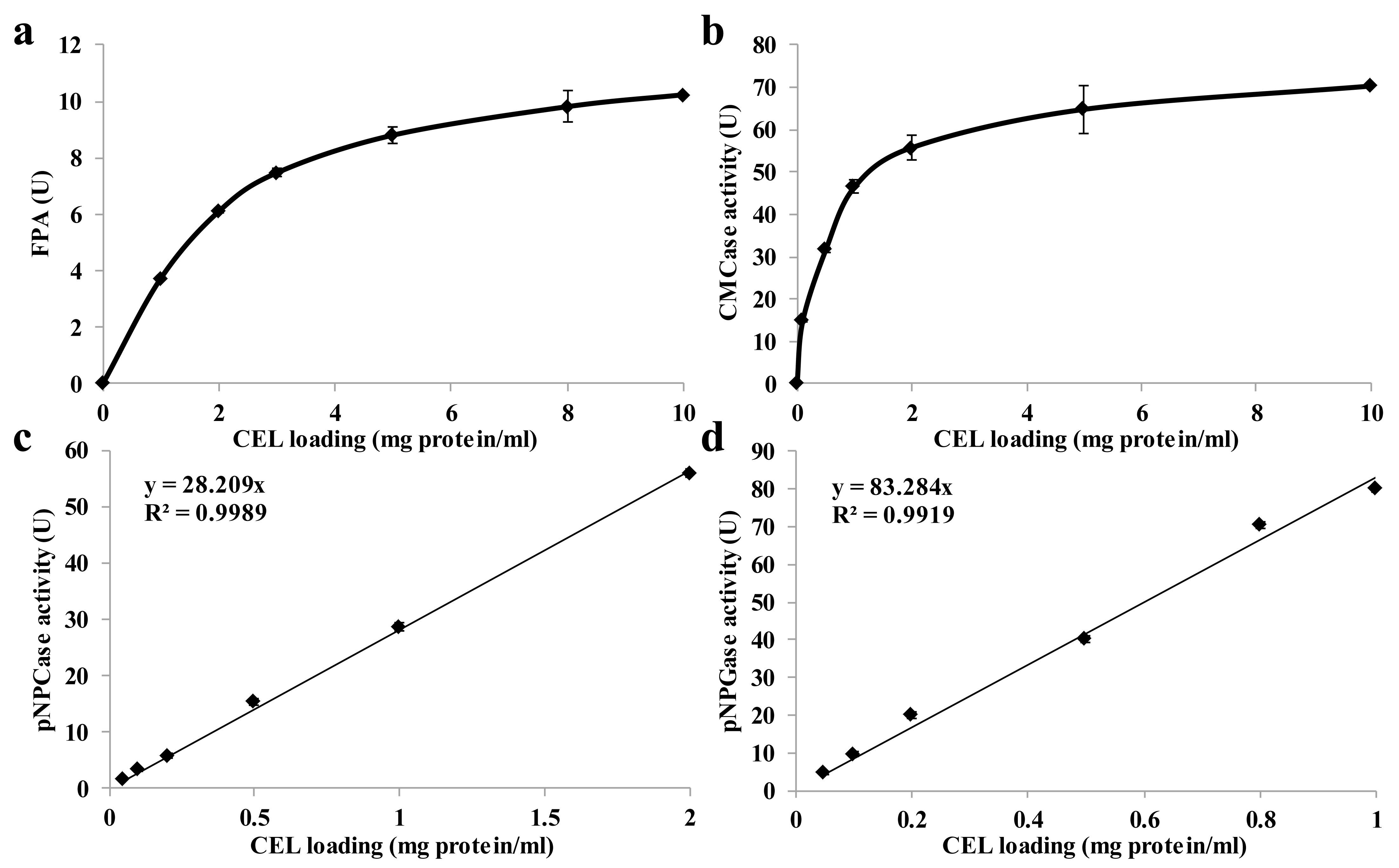

Supplement: Supplementary file 7 — Additional file 7: Figure S7. Correlation between FPA (a), activities of CMCase (b), pNPCase (c), pNPGase (d) and enzyme loading. One unit of enzymatic activity was defined as the amount of enzyme that produced 1 μmol of glucose (for FPA and CMCase) or pNP (for pNPCase and pNPGase) per minute under the conditions. [file 13068_2017_993_MOESM7_ESM.tif]
